# Stabilized Production of Lipid Nanoparticles of Tunable Size in Taylor Flow Glass Devices with High-Surface-Quality 3D Microchannels

**DOI:** 10.3390/mi10040220

**Published:** 2019-03-27

**Authors:** Peer Erfle, Juliane Riewe, Heike Bunjes, Andreas Dietzel

**Affiliations:** 1 Technische Universität Braunschweig , Institute of Microtechnology, 38124 Braunschweig, Germany; a.dietzel@tu-braunschweig.de; 2 Technische Universität Braunschweig, Center of Pharmaceutical Engineering, 38106 Braunschweig, Germany; j.riewe@tu-braunschweig.de (J.R.); heike.bunjes@tu-braunschweig.de (H.B.); 3 Technische Universität Braunschweig, Institut für Pharmazeutische Technologie, 38106 Braunschweig, Germany

**Keywords:** microfluidics, taylor flow, passive micromixing, precipitation, lipid nanoparticles

## Abstract

Nanoparticles as an application platform for active ingredients offer the advantage of efficient absorption and rapid dissolution in the organism, even in cases of poor water solubility. Active substances can either be presented directly as nanoparticles or can be integrated in a colloidal carrier system (e.g., lipid nanoparticles). For bottom-up nanoparticle production minimizing particle contamination, precipitation processes provide an adequate approach. Microfluidic systems ensure a precise control of mixing for the precipitation, which enables a tunable particle size definition. In this work, a gas/liquid Taylor flow micromixer made of chemically inert glass is presented, in which the organic phases are injected through a symmetric inlet structure. The 3D structuring of the glass was performed by femtosecond laser ablation. Rough microchannel walls are typically obtained by laser ablation but were smoothed by a subsequent annealing process resulting in lower hydrophilicity and even rounder channel cross-sections. Only with such smooth channel walls can a substantial reduction of fouling be obtained, allowing for stable operation over longer periods. The ultrafast mixing of the solutions could be adjusted by simply changing the gas volume flow rate. Narrow particle size distributions are obtained for smaller gas bubbles with a low backflow and when the rate of liquid volume flow has a small influence on particle precipitation. Therefore, nanoparticles with adjustable sizes of down to 70 nm could be reliably produced in continuous mode. Particle size distributions could be narrowed to a polydispersity value of 0.12.

## 1. Introduction

Brain diseases such as Alzheimer’s, stroke, and brain tumor, as well as mental diseases such as depression, are among the most important diseases of the human population worldwide and represent a major contributor to the global pharmaceutical market [1,2]. In order to combat such diseases, it is necessary not only to find a suitable active substance, but also to ensure a sufficiently good bioavailability of this substance by developing suitable formulations [3,4]. Despite good results during in vitro tests, formulations could often fail during in vivo experiments. In many cases, the active substances are insufficiently absorbed by the body due to poor water solubility or cannot overcome barriers such as the blood–brain barrier [5,6]. To cope with these problems, new approaches for drug delivery have been developed like pH modification, polymeric micelles, amorphous forms, or nanonization, for example, in the form of lipid nanoparticles [7]. The use of lipid nanoparticles, in which active pharmaceutical ingredients are incorporated, offers the advantages of targeted and controlled drug delivery as well as good stability and high drug incorporation, thanks to their small size and large specific surface area [6,8,9,10,11,12]. Important factors in drug release are therefore the mean size of the nanoparticles and the width of the size distribution [13,14].

Nanoparticles or nanoemulsions are already being produced on an industrial scale. State-of-the-art technology is based on the top-down method, in which previously produced particles are reduced in size by various techniques, such as high-pressure homogenization, ultrasonification, or premix membrane emulsification [15,16,17]. However, these methods can have destructive effects due to high thermal energy input and mechanical abrasive shear stress [18]. As an alternative, bottom-up methods usually based on the principle of precipitation can be used. They can create the particles in a single step requiring an effective mixing process, so that the particles assume the desired size. A typical process uses a lipid dissolved in a water-miscible organic solvent together with the active ingredient and an additional surfactant. Through mixing with an aqueous medium as an antisolvent, the solubility of the lipid in the solvent is greatly reduced and the solution becomes supersaturated, causing the nanoparticles to precipitate [19,20]. This offers the advantage of a process with low thermal and mechanical energy input, which is a decisive requirement for (thermo)labile active substances. It is crucial for the quality of the lipid nanoparticles that the mixing takes place as quickly as possible at all locations within the reactor so that the supersaturation in the system is uniform. In order to achieve small particle sizes, only primary nucleation should take place and the particles should only have a short time to continue growing after a critical nucleus is formed. To achieve this, the mixing time should be as short as possible [21]. This demands the use of miniaturized reactors with a precise control of the mass flow and mixing time as well as the establishment of a reliable device control for tunable nanoparticle size.

Mixing in microscales can provide controlled conditions due to intensified mass and heat transport and can make a significant contribution to improving the procedure. The small size of the systems enables reproducible and controlled processes. Microfluidic processes also enable the continuous production of lipid nanoparticles. However, in laminar flow conditions in straight miniaturized channels, convective mass transport perpendicular to the flow direction does not occur and mixing occurs by slower Taylor–Aris dispersion [22]. Various geometric designs for microfluidic systems were proposed to speed up the mixing, like flow-focusing or lamination mixers [23,24,25,26,27]. A further method of a more efficient mixing process is the segmentation of the continuous flow with a low miscible medium such as gas, which results in a periodic gas–liquid flow, a so-called ‘Taylor flow’ [28,29]. This type of flow suppresses free convection between liquid plugs but creates vortices within the liquid, resulting in improved convective mixing [30,31]. In this way, a quasi-batch mode process is established, in which very effective mixing occurs in a multitude of extremely small reactors in the form of liquid plugs. In contrast to continuous micromixers, in which the solutions require a longer residence time due to Hagen Poiseuille flow conditions, the Taylor flow reduces the required residence time of the solutions in the channel [32].

A further advantage of mixing in Taylor flow microsystems is that plug sizes can be adjusted by a simple change of the volume flows of gas and liquid, which can be optically monitored in a transparent device [33]. In this previous work, a microfluidic system with symmetrical inflow design was demonstrated to be an efficient and well-controllable Taylor flow mixing device. Femtosecond laser fabrication was used as a fast and precise fabrication process, which allows realizing channels with varying depths and with circular cross-sections that cannot be produced using more conventional fabrication techniques. Further, the use of glass as an inert material seems preferable over Polydimethylsiloxane (PDMS), which is the material used in most other works on micromixers. It could be shown that mixing times in Taylor flows can be reliably controlled during the precipitation processes, so that the size of the lipid nanoparticles can be tuned [32,33,34,35]. However, glass-based 3D manufacturing requires research in new microstructuring processes.

Despite these advancements, current Taylor flow devices are susceptible to fouling occurring at the channel walls, which up to now has impeded a broad investigation of nanoparticle formation over a wide range of Taylor flow conditions. Therefore, advances in channel wall treatments for smoother surfaces are necessary. To address current shortcomings in glass microfabrication as found in earlier work, novel procedures for improved glass surface properties to reduce problematic fouling have to be developed. Only when fouling can be suppressed can a wide range of stable volume flow rates for the liquid and gas phase as well as for different concentrations of the organic phase be investigated which is required for studying how nanoparticle properties can be tuned.

## 2. Materials and Methods

### 2.1. Segmented Gas–Liquid Flow

**Figure 1:**
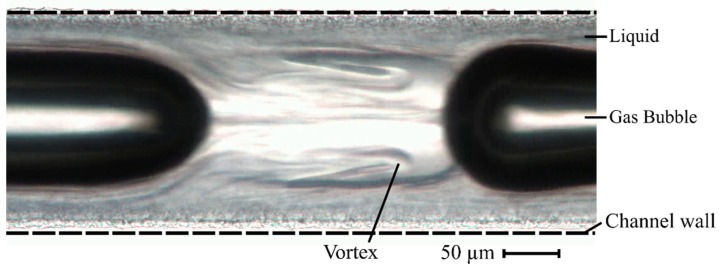
A microscopic view of a transparent microchannel showing vortices in the segmented gas–liquid flow. The wall friction in the channel causes a relative backflow of the liquid so that it circulates symmetrically around the central axes of the channel in the form of vortices. The bubble diameter is lower than the channel diameter (dashed lines indicate channel wall positions) by the amount of 2δ.

In gas–liquid flows in microfluidic systems, where surface forces and viscous forces dominate over gravity and inertia, different types of flow can occur depending on the respective gas and liquid phase velocities, which can be classified into six categories: bubbly flow, Taylor flow, Taylor-annular flow, annular flow, churn flow, and dispersed flow [36,37]. If the flow rates of the gas and liquid phases are in the same range, the Taylor flow is formed, in which the gas phase in the form of bubbles interrupts the liquid flow, which forms regular plugs. The periodic bubbles are surrounded by a thin liquid film and typically show a length which is larger than the channel diameter. In the plugs, vortices, which run symmetrically to the central axes of the circular channel, are generated by a relative backflow of liquid which, depending on the flow parameters, causes a different mixing performance (Figure 1) [38,39]. The capillary number Ca can be used as the indicator for the occurrence of vortices:

(1) $$Ca=\frac{\mu \cdot U_{b}}{\gamma }$$

where *µ* and γ represent the viscosity and surface tension of the liquids, respectively, and $U_{b}$ represents the gas bubble velocity [40]. Taylor flow will turn into the annular flow at increasing values of the capillary number. In practice, Ca < 0.7 should be selected for obtaining Taylor flows [41]. The gas bubbles are not in direct contact with the channel wall, but are separated by a thin film of liquid. The thickness δ of the thin film between the bubbles and the channel walls depends on the channel radius r and Ca as can be described with:

(2) $$\frac{\delta }{r}=\frac{1.34\cdot Ca^{2/3}}{1+2.5\cdot \left(1.34\cdot Ca^{2/3}\right)}$$

This relation was found based on experiments with optical measurements of film thickness [40]. The occurrence of Taylor flow does not necessarily cause the formation of Taylor vortices. At small capillary numbers, δ will be small compared to r and relatively large vortices are generated, whose centers are located closer to the channel wall, while with increasing gas bubble velocity and thus also rising capillary number, the vortices become smaller and their centers shift towards the central axis of the channel. If the capillary number increases further, δ will no longer be much smaller than r, almost the entire liquid flows around the bubble, and no vortices occur. An increase in the gas volume flow will increase the flow velocity and at the same time shorten the liquid plugs whereby vortices decrease in size but vortex velocities increase [42,43]. As a consequence, the mixing is intensified when compared to larger liquid plugs [33,44].

### 2.2. Microfluidic Design

**Figure 2:**
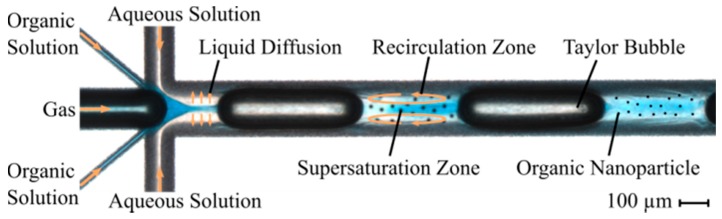
A microscopic image of the microfluidic system with initial flow-focusing and subsequent Taylor flow. On the left side of the image, five channels join at an intersection to enter the main channel in which the mixing process within the Taylor flow takes place. Through the middle inlet channel, the gas phase flows into the main channel. First, the channels with the organic phase and then the channel with the aqueous phase reach the intersection symmetrically from above and below the gas channel. The organic phase is colored with methylene blue for better visibility during the mixing process. The orange arrows illustrate the liquid flow in the inlet channel, the diffusion of the organic solvent into the aqueous solution, and the movement of the Taylor vortices within a plug. During the mixing process, the organic lipid supersaturates in the solvent and precipitates as nanoparticles, illustrated as black dots in the main channel.

A microfluidic design with axis-symmetrical channels and a combined intersection (Figure 2), containing the flow-focusing interface and the Taylor flow formation, was chosen based on an earlier work [33]. This system comprises a total of three inlets for the aqueous phase, the organic phase, and the gaseous phase. The aqueous and organic inlets divide into two channels, which join again at the intersection, while the gas channel directly enters the intersection. The main channel, in which the mixing takes place in a Taylor flow, exhibits a hydraulic diameter of 193 µm and a total length of 21 mm. Inlets for the gas phase and the aqueous phase exhibit hydraulic diameters of 146 µm and 87 µm, respectively. We also wanted to study the influence of the organic phase inlet diameter, because there were first indications that it has influence on the fouling occurring at the walls of the main channel during ongoing precipitation. A first and a second design variant exhibit a hydraulic diameter of the organic phase inlet of 58 µm and 29 µm, respectively. A third variant differs from the second variant in that it underwent additional tempering at 760 °C for one hour.

### 2.3. Fabrication Process

Borofloat^®^ 33 borosilicate glass from Schott (Mainz, Germany), available in the form of wafers, was used as the material for the microfluidic systems. A femtosecond laser microstructuring system (microSTRUCT c; 3D Micromac AG, Chemnitz, Germany) was employed to structure the half sides of the microchannels in the glass wafer. The system is equipped with a Yb:KGW (Ytterbium-doped potassium–gadolinium–tungstate) laser source, which emits linearly polarized light at the fundamental wavelength of 1030 nm with an output power of 15 W. The focus of the laser was delivered to the glass substrate through an f-theta lens with a 100 mm focal length to a spot with a diameter of 18 µm exhibiting a Gaussian intensity distribution. Pulses of 212 fs were emitted at a frequency of 600 kHz. To realize a high processing speed, the laser beam was positioned with a galvanometer scanner (Scanlab RTC5, Puchheim, Germany). The pulse energy in the laser spot was 12.85 µJ, while the speed of the laser spot moving on the substrate was set at 2000 mm/sec. The displacement strategy of the laser spot was based on a parallel line pattern with line spacing of 4 µm and an offset to the nominal channel edge of 4 µm. Each of the following scan layers was rotated by 30° against the previous one. This scanning strategy homogenized the removal of glass material and prevented periodic surface structures resulting from simpler scanning patterns. A single line pattern produces an ablation depth of 8.33 µm, so that a total of 12 layers was used to reach 100 µm in the center of one channel half. The depth was measured with a laser-scanning microscope (VK-X260K, Keyence, Osaka, Japan). By focusing the laser on the surface, the laser intensity was increasingly cut off at the edge of the structured to the unstructured area with increasing depth. As a result, a tapered channel wall starting at 64° was created, which smoothly merged into a flat channel bottom. This resulted in an almost semicircular cross-section. A second glass wafer was structured with mirrored microchannels. The wafers were dipped for 90 s in a glass-etching solution (phosphoric acid, hydrofluoric acid, and water, mixed 20:6:9) to remove the glass particles and filaments. The wafers were cleaned with a high-pressure jet and Caro’s acid (sulfuric acid with hydrogen peroxide 1:1) before they were thermally bonded together at 630 °C under a pressure of 4.9 kPa for six hours. Only microsystems of the third design variant were heated to 760 °C (slightly above the melting temperature of 680 °C) for one hour. Induced changes of the channel surfaces were optically analyzed using a scanning electron microscope (Phenom XL, ThermoFischer Scientific, Waltham, MA, USA). In addition, the roughness of the channel surfaces was determined with a laser-scanning microscope (VK-X260K, Keyence, Osaka, Japan).

### 2.4. Chemicals and Solutions

For the organic phase, 5 mg/mL castor oil (D-Bremen, Henry Lamotte Oils, Bremen, Germany) as lipid and 2.5 mg/mL polysorbate 80 (D-Steinheim, Sigma-Aldrich, St. Louis, MS, USA) as surfactant were dissolved in ethanol. The surfactant has the function to stabilize the lipid nanoparticles after precipitation and to prevent coalescence. Deionized water was used for the aqueous phase and nitrogen was used for the gaseous phase. The aqueous solution was filtered with a 0.2 µm hydrophilic syringe filter (Minisart^®^ NML 16534-K) and the lipid solution with a 0.2 µm hydrophobic syringe filter (Minisart^®^ HY 16596-HYK).

### 2.5. Precipitation Process

The solutions and the gas were injected via three syringe pumps (Nemesys Base120 + Low-Pressure modules). In the experiments, the overall liquid flow rate was set at 200 µL/min. For each volumetric flow rate of the liquid phase, the gas flow rate was increased successively from 25 µL/min, 31 µL/min, 55 µL/min, 75 µL/min, 130 µL/min, 350 µL/min, and 550 µL/min to 900 µL/min. The ratio of the organic phase to the total liquid flow rate was set to 10%, 20%, and 30%. For each volumetric flow rate of the gas phase and for each investigated concentration in the organic phase, three precipitation experiments were carried out and collected in separate Eppendorf Safe-Lock Tubes.

### 2.6. Analysis of Segmented Gas–Liquid Flow

The Taylor flow was optically observed with a microscope (Zeiss Model Axio Vert.A1) equipped with a camera (Basler Model acA 2040-90uc) that records videos. Using a specially developed image-processing algorithm in Matlab^®^, all gas bubbles and plugs in each frame of the video were automatically detected [33]. The algorithm recognizes each gas bubble in the binary image and measures its size and position in the channel, from which the lengths and positions of the plugs are calculated. With a conversion factor, the sizes of pixel units were converted into metric units. This process was performed for each frame in the video, resulting in an average size of the gas bubbles and plugs, together with standard deviations. This method provided the possibility of a direct correlation between the length of the plugs and the size of the nanoparticles, which was determined during the precipitation process.

### 2.7. Particle Size Measurement

Particle characterization was performed using dynamic light scattering (DLS, Horiba Model SZ-100) to determine the z-average (mean particle diameter) and polydispersity index (PDI). Four measurements of 3 min each were performed, where the first measurement was discarded because of the ongoing stabilization of the sample.

## 3. Results and Discussion

**Figure 3:**
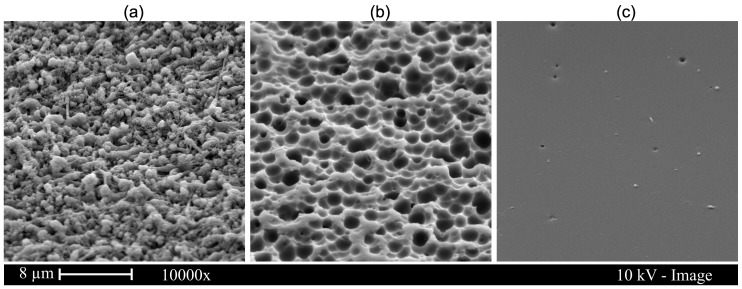
SEM images of laser-structured microchannel surfaces after certain steps of the fabrication. The first (**a**) image shows the surface of the glass right after the laser ablation. For the second image (**b**), the glass was dipped in a glass-etching solution for 90 s resulting in typical surface pits. For the third image (**c**), the etched glass was tempered at 760 °C for 1 h resulting in an almost perfectly smooth surface.

Directly after laser ablation, the channel surfaces showed a roughness and a covering with glass filaments and particles (Figure 3a). The subsequent etching process improved the surface quality, resulting in a more uniform channel wall surface covered with round pits with a diameter of up to 2 µm (Figure 3b).

**Figure 4:**
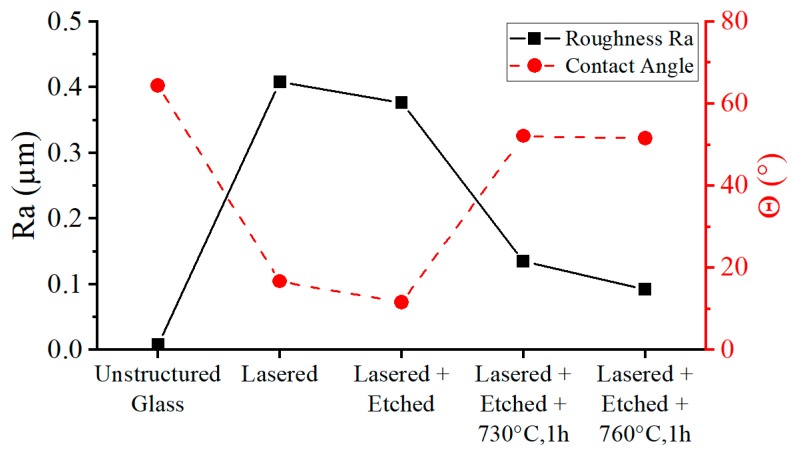
The roughness and contact angle values on glass surfaces measured after the individual glass treatment steps.

The tempering of microsystems of the third design variant further smoothened the channel surface. The high temperatures caused the glass to flow as a highly viscous liquid. The etch pits (Figure 3c) increasing the channel surface area were reduced during melt flow by the effect of surface tension, which smoothed the channel walls. The areas with the most pointed geometry experienced the highest surface tension force. The roughness was reduced from *Ra* = 0.41 µm, measured after laser ablation and etching, to *Ra* = 0.09 µm, measured after thermal treatment (Figure 4). The initial roughness of an unstructured and untreated glass wafer is *Ra* = 0.004 µm. Furthermore, the contact angle θ was measured on the glass surfaces at the individual process steps. A surface roughness (as induced by laser ablation) caused a change of the contact angle to $\cos\theta^{*}=r\cdot \cos\theta$, with r as the ratio of the actual area to the projected area. As can be seen in Figure 4, the ablation-induced roughness amplified the hydrophilicity of the surface, resulting in a decrease in the contact angle [45]. The annealing reduced the ratio of the projected surface to the actual surface, which resulted in an increase in the contact angle towards the values of unstructured glass.

**Figure 5:**
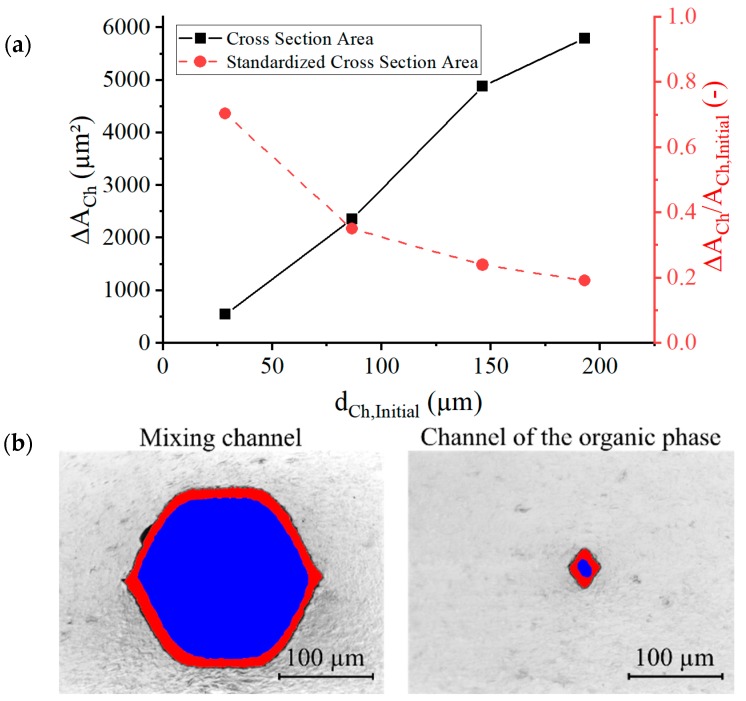
(**a**) The measured absolute reduction of the cross-section area $\Delta A_{Ch}$ and the relative reduction of the cross-section area $\Delta A_{Ch}/A_{Ch,Initial}$, dependent on initial channel diameters $d_{Ch,Initial}$ by thermal treatment. (**b**) A microscopic cross-sectional view of two microchannels. The left image shows the mixing channel and the right image shows the smaller channel for the organic phase. The channel cross-sectional areas are colored to show the difference between before (red area) and after (superimposed blue area) the thermal treatment.

During the tempering of the microsystem, a radial shrinkage of channel cross-sectional areas was also observed. Changes in the cross-sectional area $A_{Ch}$ measured before and after the annealing are shown in Figure 5. The absolute decrease $\Delta A_{Ch}$ was more apparent in microchannels with a larger diameter, but the relative shrinkage $\Delta A_{Ch}/A_{Ch,Initial}$ was more pronounced in narrower channels. For the narrowest channel (organic phase inlet), the cross-sectional area was reduced to 30% of its initial value (from 29 µm to 16 µm).

**Figure 6:**
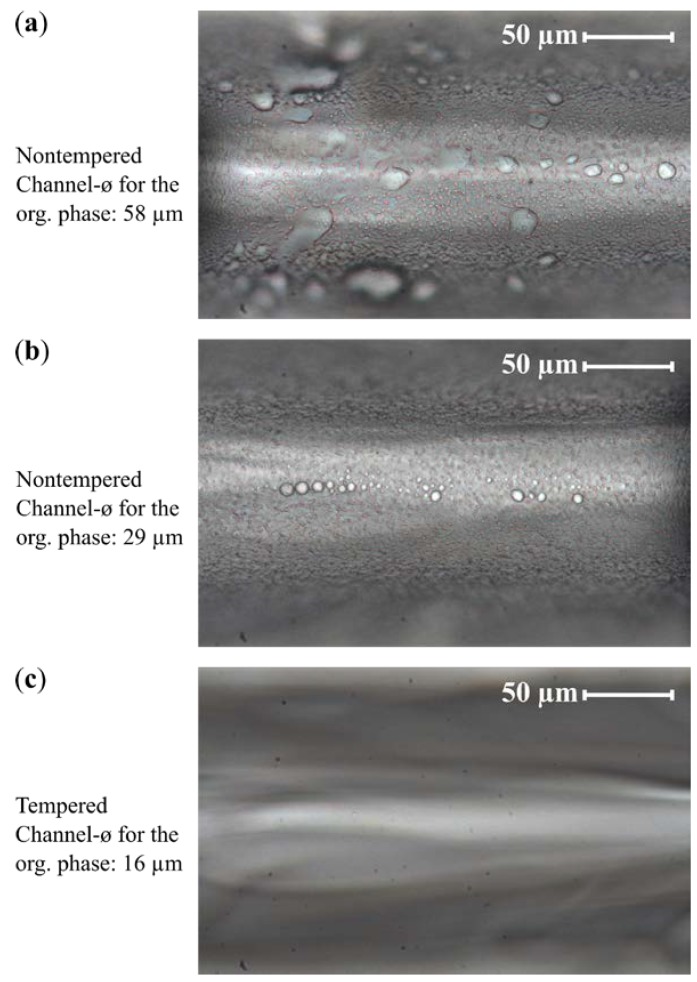
Microscopic images of the main channels of three microfluidic systems taken after 2 min of continuous operation at positions 40 µm after the intersections. The first microsystem (**a**) was made with an organic phase inlet channel of nominally 58 µm in diameter and the second microsystem (**b**) of nominally 29 µm. The third microsystem (**c**) had a nominally 29 µm inlet channel diameter before further treatment was tempered at 760 °C for one hour; after that it exhibited an organic phase inlet channel of 16 µm in diameter and smoothed channel walls. In the first microsystem, strong fouling occurred, while in the second microsystem, the fouling was decreased due to more focused injection, but small droplets were still found. In the tempered microsystem, no droplets could be found on the channel walls. The small black dots in the images are background dust particles (they appear identically in all three images).

Figure 6 shows the degree of fouling in the form of castor oil droplets accumulating at the channel wall, observed with the three design variations. Due to a decreasing diameter of the inlet channel (from a to b) for the organic phase, a more focused injection of the organic phase into the center of the main channel was provided. This caused improved mixing of the solutions before the organic phase was exposed to the channel wall. Furthermore, a reduced surface roughness of the channel wall (c) prevented the oil droplets from being deposited in the cavities on the channel wall, thus serving as nucleation points for further fouling. Fouling cannot be completely eliminated, but smoothing the surface prevents the permanent adhesion of the droplets on the channel wall and allows the long-term operation of the microdevices. Only the second and third design variants could be used in the following for the investigations of the flow behavior of the Taylor flow and the precipitation processes.

**Figure 7:**
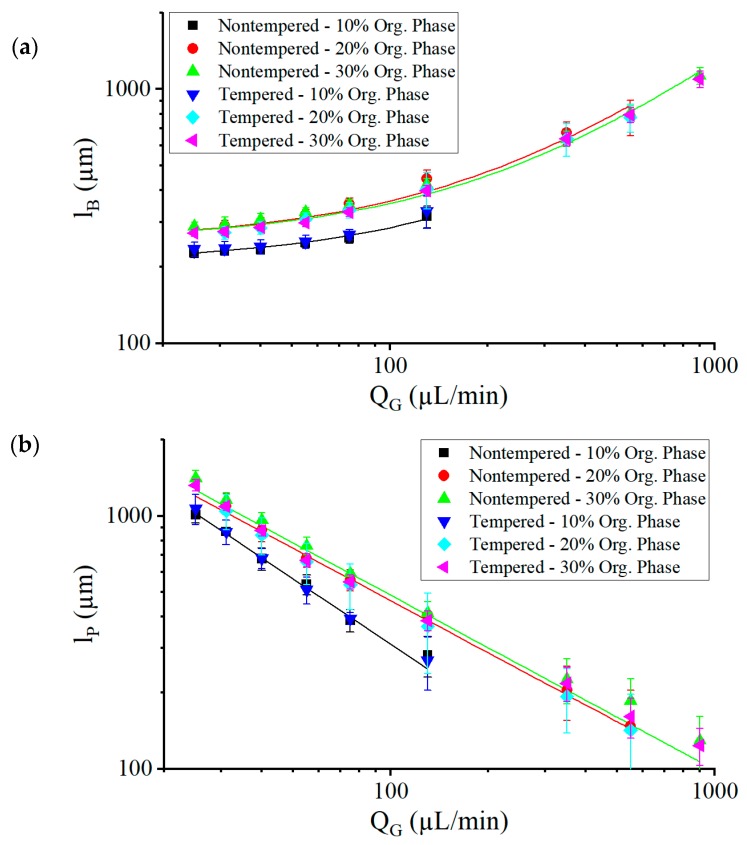
The lengths of the gas bubbles $l_{B}$ (**a**) and plugs $l_{P}$ (**b**) obtained in the experiments with nontempered and tempered systems as a function of the gas volume flow $Q_{G}$ at a total liquid volume flow $Q_{L}$ of 200 µL/min. The trend lines are shown simply to guide the reader’s eye.

With design variants 2 and 3, the Taylor flow in the selected volume flow ranges can be controlled precisely and reproducibly, so that stable plug generation can be realized with plug lengths ranging between half the channel diameter and seven times the channel diameter. Figure 7 shows the plug and gas bubble quantities as a function of the gas volume flow rate. Within the measured range, the size of the gas bubbles $l_{B}$ increased proportionally with the factor m, with an increasing gas volume flow rate $Q_{G}$ according to:

(3) $$l_{B}=m\cdot Q_{G}+l_{B0}$$

where $l_{B0}$ is the minimum bubble size for a system having the total liquid flow rate $Q_{L}=Q_{AP}+Q_{OP}$. $Q_{AP}$ and $Q_{OP}$ each represent the liquid volume flow rate of the aqueous and organic phases. The size of the plugs $l_{P}$, on the other hand, decreased antiproportionally to the gas volume flow $Q_{G}$. A reduction in the concentration of the organic phase led to increasing instability of the Taylor flow. As the concentration of the organic phase decreased, the range of gas volume flow rates for the stable Taylor flow began to decrease. At 20% of the organic phase, the Taylor flow broke down at a gas volume flow of $Q_{G}$ = 25 µL/min, while at $Q_{G}$ = 900 µL/min an annular flow was generated. At 10% of the organic phase, the regime of the stable Taylor flow decreased further, to a range between 25 µL/min and 200 µL/min. Above 200 µL/min, a nonperiodic gas bubble break occurred. A possible cause could be the effects of interfacial tension or volume contraction as they destabilize the mixing system more strongly with small proportions of the organic phase. At a lower concentration of the organic phase, the phase boundary relative to the volume ratio of the aqueous phase to the organic phase was greater than at a higher concentration of the organic phase. As a result, additional convection could be induced, due to gradients in surface tensions. These currents are also called Marangoni flows [46]. With the higher ratio of the organic phase, this effect was less pronounced in relation to each phase.

The Taylor flows in the nontempered and tempered systems showed similar characteristics of the gas bubble tear-off. In both cases, gas bubbles of the same length flowed into the mixing channel. On the other hand, the plugs in the tempered system had a slightly shorter length compared to the nontempered system. At a concentration of 20% and 30% of the organic phase, the plug sizes of the tempered system were on average 5% and 8% smaller, respectively, than those of the nontempered system. At a concentration of 10% of the organic phase, the plug sizes of the tempered system did not show a significant difference compared to the nontempered system. A possible cause could be the shrinking of the channels after tempering, where the mixing channel was reduced in diameter by 10%. This resulted in a larger ratio of channel surface to channel cross-section, so that more surface area for the thin film was available than volumes for the plug. There was proportionally more surface area for the thin film leakage, so that less volume flowed through the channel as a plug. Correspondingly, the ratio of measured to calculated plug length in the tempered microsystem decreased further than in the nontempered microsystem.

**Figure 8:**
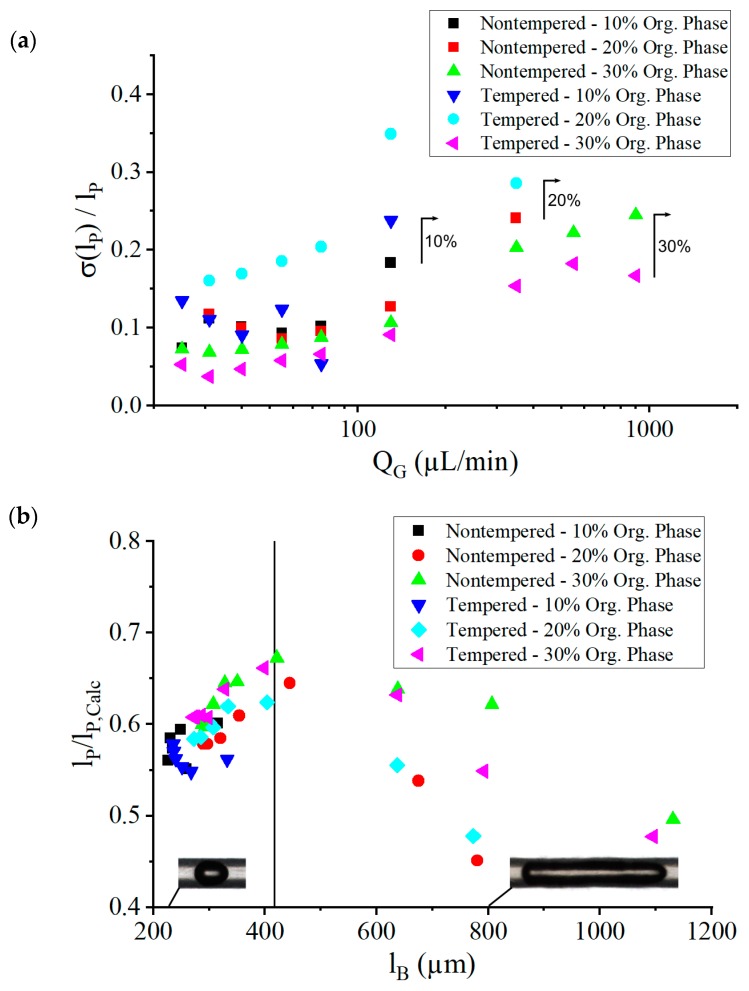
(**a**) The standard deviation of the plug length normalized to the average plug length dependent on the gas volume flow rate $Q_{G}$. A steep increase is observed at $Q_{G}$ = 130 µL/min. The three marking lines define the onset of Taylor flow instability, with increasing gas volume flow rate $Q_{G}$ at the respective concentrations of the organic phase. (**b**) Ratio of optically measured plug size to the plug size calculated with Equation (4) without considering thin-film leakage, dependent on the bubble size $l_{B}$. The total liquid volume flow rate was kept at 200 µL/min. The vertical line at $l_{B}=2d_{Ch}$ marks the transition from almost round bubbles to elongated bubbles, at which point $l_{P}/l_{P,Calc}$ reached a maximum for 20% and 30% concentrations of the organic phase $l_{B}=2d_{Ch}$. This transition occurred at $Q_{G}$ = 130 µL/min (see also Figure 9).

Even though the Taylor flows seemed to produce very regular flow patterns, the gas bubble and plug formation did not occur absolutely periodically. Figure 8a shows the relative standard deviation of the plug size (measured in absolute standard deviation obtained from optical images normalized to the measured length of the plug size), dependent on the gas volume flow $Q_{G}$. As the gas volume flow increased, the fluctuations also increased. At $Q_{G}$ = 130 µL/min, a rather sharp increase of fluctuations was observed. This resulted in deviations in the mixing time of the solutions, because mixing time depends linearly on the plug length [33].

Neglecting the thin film between the bubbles and the channel walls, the plug size can be calculated with the gas volume flow rate and the liquid volume flow rate [33] as:

(4) $$l_{P,Calc}=\frac{Q_{L}}{Q_{G}}l_{B0}+Q_{L}m$$

which results in values larger than the measured plug length $l_{P}$. Due to a liquid film of thickness δ≠0 at the channel wall, some of the solution did not participate in plug formation. Figure 8b shows the ratio of optically measured plug sizes to the plug sizes calculated $l_{P}/l_{P,Calc}$, dependent on gas bubble length $l_{B}$. At low values of $l_{B}$, which are smaller than $2d_{Ch}$, gas bubbles also decreased in diameter, resulting in a thicker fluid film at the wall. At increasing values of $l_{B}$, the ratio $l_{P}/l_{P,Calc}$ reached a maximum, but then decreased with increasing $l_{B}$ as a consequence of the increasing speed of the gas bubbles $U_{B}$, which increased the film thickness δ according to Equation (2), but also because the longer gas bubbles offered a larger coating volume for a given δ. The best conditions, in terms of plug monodispersity and low thin-film leakage, were therefore found at $l_{B}\leq 2d_{Ch}$.

**Figure 9:**
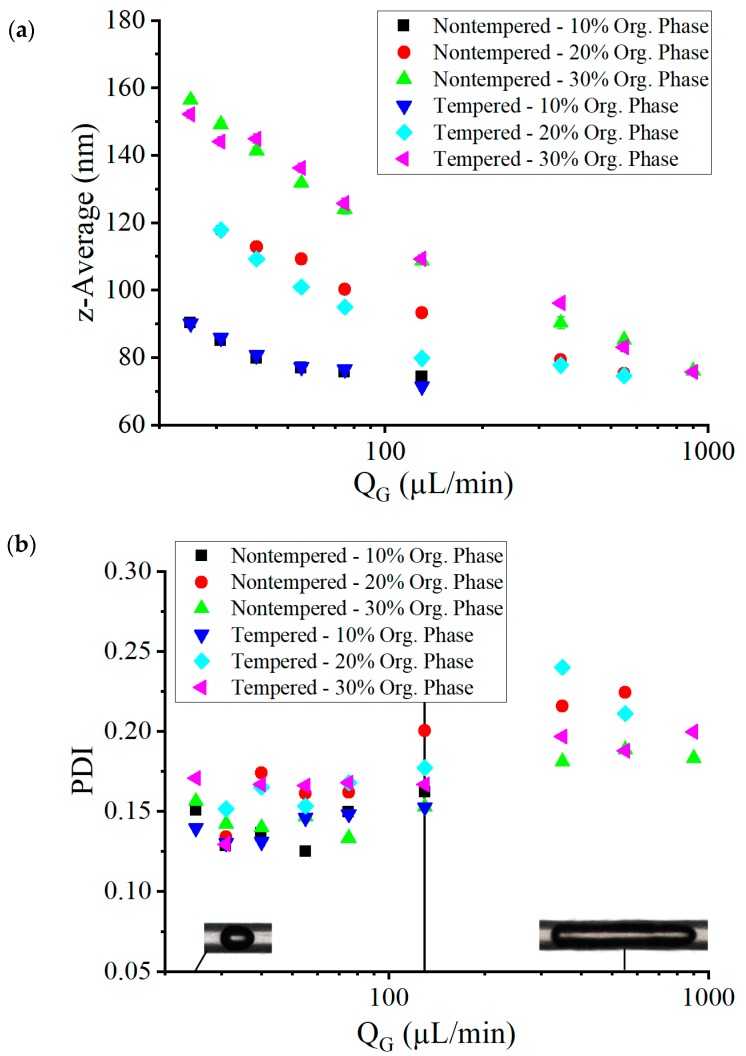
The average size (**a**) of lipid nanoparticles and their polydispersity index (PDI) (**b**) depending on the gas volume flow rate in the nontempered and tempered system at a total liquid volume flow rate of 200 µL/min and different concentrations of the organic phase. The vertical line at $Q_{G}$ = 130 µL/min marks the point of lowest difference between $l_{P}$ and $l_{P,Calc}$ at $l_{B}\approx 2d_{Ch}$ as indicated in Figure 8b.

If the gas volume flow is increased, a significant decrease in the mean particle size (determined by DLS) could be observed for all tests carried out (see Figure 9a). Even at low gas volume flow rates, small particle sizes of less than 160 nm were achieved. In this area, a stable Taylor flow was observed during the experiments. As the gas flow rate increased, the frequency of the liquid plugs increased, while the size of the plugs decreased. The convection induced by the Taylor flow thus took place in much smaller volumes, which intensified mixing and accelerated the precipitation process. The concentration of the organic phase had a strong influence on the average particle size. Significantly, smaller average particle diameters were achieved at low ethanol concentrations. This corresponds to Schubert’s investigations, in which significantly smaller particle sizes were observed for lower amounts of the added lipid-containing phase [19]. The most likely cause for this was the change in the phase boundary between the organic and aqueous solution. Due to the lower ratio of the organic phase flow to the total liquid flow, the organic phase became more focused in the mixing channel, with a higher ratio of surface area to volume. This led to an increased diffusion in the Taylor vortices and, as such, accelerated mixing.

The values of the nanoparticle polydispersity index (PDI; also measured with DLS) are shown in Figure 9b. The PDI value for all set parameters was between 0.12 and 0.24. For small gas volume flow rates below 75 µL/min, small PDI values of around 0.15 were observed, with the lowest PDI values being achieved at a concentration of the organic phase of 10% in the nontempered and tempered system. This represents particle size distributions similar to the ones achievable with established process techniques, such as high-pressure emulsification [47,48]. For low gas volume flow rates, the concentration of the organic phase had no statistically relevant influence on the particle size distribution. The PDI varied around a value of 0.15 at low gas volume flow rates $Q_{G}$, before it began to increase at a volume flow of 130 µL/min, which marked the point where $l_{B}\approx 2l_{Ch}$ (see maximum in Figure 8b) led to the increasing thickness of the thin film δ with increasing gas bubble velocity $U_{B}$. This led to the situation that part of the solutions was not involved in the fast Taylor mixing process but was mixed more slowly due to the thin film. This could explain the higher probabilities of larger particles beside smaller particles being created in Taylor mixed volumes.

**Figure 10:**
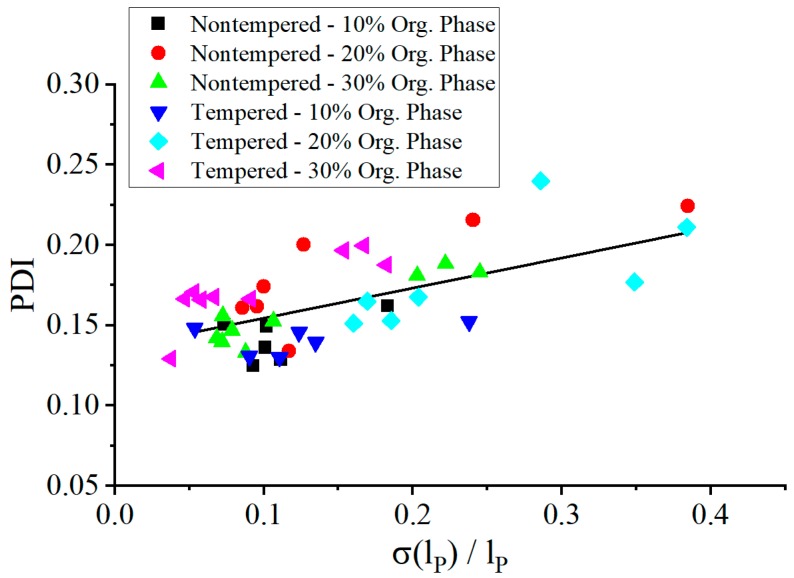
The polydispersity index (PDI) dependent on the standard deviation of the plug length normalized by the average plug length. The trend line was calculated from all measuring points and only serves as a guide for the eye.

The broadening of the particle size distribution correlated with plug size variations as shown in Figure 10, where the nanoparticle PDI was given in dependence on the relative plug length variation. At more stable plug sizes, the mixing time was more reproducible and the PDI values reduced.

The fluids were continuously injected at the intersection before entering the mixing channel caused by the constant flow rate of the syringe pumps. In the case of high gas volume flows or long gas bubbles, when a gas bubble entered the mixing channel, the aqueous and organic phases continued to be injected into the channel but were pressed against the channel wall. This happened until a bubble break occurred and the liquid could flow in the form of a plug where it was mixed by the Taylor vortices. This scenario resulted in poorer mixing of the solutions. The gas volume flow rate should be selected, depending on the liquid volume flow rate used, as this was the only way to achieve optimum particle sizes and PDI values.

**Figure 11:**
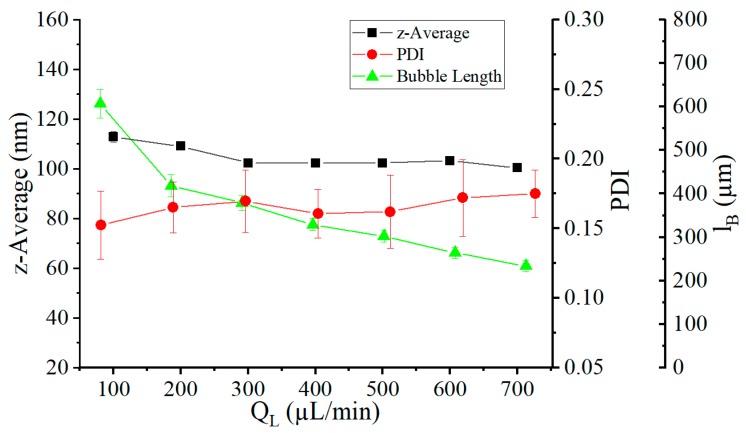
The average size of the nanoparticles and their polydispersity index (PDI) with a fixed plug size of 400 µm and a total liquid flow rate of 100 µL/min to 700 µL/min in the nontempered system. The concentration of the organic phase to the liquid volume flow was 30%.

As can be seen in Figure 11, the liquid volume flow rate $Q_{L}$ had almost no influence on the particle sizes. The volume flow rate $Q_{L}$ was varied in a range from 100 µL/min to 700 µL/min in 100 µL/min steps, while the concentration of the organic phase was fixed at 30%. The gas volume flow rate $Q_{G}$ was variably set to a value so that a constant plug size of 400 µm was achieved, wherein the size of the gas bubbles decreased with each increase in the liquid volume flow rate. For the same gas volume flow rates and larger liquid volume flow rates, slightly smaller particles were produced (reduced from 112 nm to 100 nm). The low influence of the liquid volume flow rate on the mean particle size was in line with our earlier works, which demonstrated the independence of the mixing speed from the liquid volume flow rate [33]. This allowed the use of such systems for the precipitation of nanoparticles at high flow rates. In contrast, an increased liquid volume flow rate led to an increased PDI from 0.15 to 0.18. The increasing velocity of the gas bubbles caused a growth of the thin film at the channel wall (Equation (2)), whereby an increased thin-film leakage of the liquid could take place.

Table 1 shows values for z-average and PDI of the nanoparticles at all organic concentrations and at three different volume flows of the gas phase as obtained after 150 days after production compared to initial values (0 day). While the particles are stable over time at an organic concentration of 10%, the particle size has increased on average by 19 nm at a 20% concentration. However, it shows that the PDI decreased by an average of 0.04. At a 30% concentration of the organic phase, average particle sizes increased by a factor of 20 or more and dispersities of over 0.4 were observed. Coalescence and Ostwald ripening are probably proceeding over time. The latter occurs when the lipid dissolves in sufficient quantity in the aqueous phase. As a result, larger particles grow stronger, while smaller particles increasingly decompose [49]. For improving long-term stability, a removal of the solvent seems necessary.

## 4. Conclusions

Microfluidic Taylor flow mixers with defined channel cross-sections were produced from inert glass by femtosecond laser processing. A tempering process was developed, which successfully reduced ablation-related roughness and hydrophilicity in the channels almost to the values of untreated glass surfaces. The channels were even given a rounder shape during the tempering process, with the channel diameters shrinking slightly. Fouling could be minimized by such smoothing of the channels and by reducing the inlet channel diameters for the organic phase. In the precipitation experiments, the lipid was dissolved in ethanol. With organic phase proportions of 10%, mean particle diameters of 70 nm to 90 nm could be achieved. It could be shown that the proportion of the organic phase had the largest influence on the particle size distributions. Quite narrow particle size distributions were obtained, characterized by PDI values of 0.12 to 0.15. For small concentrations of the organic phase and high gas volume flow rates, however, the resulting Taylor flow was unstable and a clear minimum of achievable particle sizes at $Q_{G}$ = 130 µL/min could be determined. For higher concentrations of the organic phase from 20% to 30%, no destabilization of the Taylor flow could be observed for the investigated gas volume flow rates between 550 µL/min and 900 µL/min. The stabilization of Taylor flow formation and thin-film leakage were found to be the important factors limiting the further reduction of the PDI beyond $l_{B}=2d_{Ch}$. It was found that short mixing times did not necessarily lead to smaller PDI values and smaller particles if the backflow in the system was large or the system became unstable. The liquid volume flow played a minor role in the particle size distribution. Higher liquid volume flow rates led to slightly smaller mean particle diameters, but also increased the PDI values. The ratio of $Q_{G}/Q_{L}$ was decisive for the stability of the Taylor flow. The precipitated nanoparticles exhibited long-term stability over 150 days as long as the solvent concentration did not exceed 10%. Future efforts to narrow the particle size distribution will focus on stabilizing the break-up into gas bubbles at small liquid plug sizes, so that even more uniform Taylor flows can form. Through the successful realization of a stabilized precipitation process by the use of liquid–gas segmented flow, subsequent investigations of the used chemical formulations can be carried out. This applies in particular to the influence of the concentrations of the lipids and surfactants used. With this approach, precipitation in Taylor flows staying below the critical point of $l_{B}\approx 2d_{Ch}$ where smallest dispersities are observed could potentially produce even smaller particles. In summary, with the presented new Taylor flow devices for stabilized production of nanoparticles, the basis has been laid for further investigating device geometries, surfactant concentrations, and other lipids in reliable experiments.

## Figures and Tables

**Table 1:** Results of long-term stability investigation. For three different volume flow rates of the gas phase and for all concentrations of the organic phase, DLS particle measurements directly after production and 150 days later are compared. Values for average particle size and PDI are given.

Gas Volume Flow Rate *Q_G_*	z-Average (nm)/PDI					
	10% Organic Phase		20% Organic Phase		30% Organic Phase	
	0 days	150 days	0 days	150 days	0 day	150 days
31 µL/min	86/0.13	88/0.14	118/0.15	139/0.11	144/0.17	3530/0.77
55 µL/min	77/0.15	80/0.14	101/0.15	125/0.11	136/0.17	2627/0.44
130 µL/min	71/0.15	76/0.18	80/0.18	104/0.10	109/0.17	3051/0.82
